# Co-culture of a Novel Fermentative Bacterium, *Lucifera butyrica* gen. nov. sp. nov., With the Sulfur Reducer *Desulfurella amilsii* for Enhanced Sulfidogenesis

**DOI:** 10.3389/fmicb.2018.03108

**Published:** 2018-12-13

**Authors:** Irene Sánchez-Andrea, Anna Patrícya Florentino, Jeltzlin Semerel, Nikolaos Strepis, Diana Z. Sousa, Alfons J. M. Stams

**Affiliations:** ^1^Laboratory of Microbiology, Wageningen University, Wageningen, Netherlands; ^2^Laboratory of Systems and Synthetic Biology, Wageningen University, Wageningen, Netherlands; ^3^Centre of Biological Engineering, University of Minho, Braga, Portugal

**Keywords:** acidophilic sulfur reduction, glycerol, 1, 3-PDO, *Lucifera butyrica*, *Desulfurella amilsii*, co-culture

## Abstract

Biosulfidogenesis can be used to remediate low pH and high metal content waters such as acid mine drainage and recover the present metals. The selection of a cheap electron donor for the process is important for the economic viability. In this work we isolated a novel versatile acidotolerant fermentative bacterium (strain ALE^T^) that is able to use a great variety of substrates including glycerol. Strain ALE^T^ is an obligate anaerobe, and cells are motile, rod-shaped, spore-forming, and stain Gram-positive. Growth occurred in a pH range from 3.5 to 7 (optimum 5.5), and temperature range from 25 to 40°C (optimum 37°C). It grows by fermentation of sugars, organic acids and glycerol. It has the ability to use thiosulfate, iron and DMSO as electron acceptors. Its genome is 4.7 Mb with 5122 protein-coding sequences, and a G+C content of 46.9 mol%. Based on 16S rRNA gene sequence analysis, the closest cultured species is *Propionispora hippei* (91.4% 16S rRNA gene identity) from the *Sporomusaceae* family (*Selenomonadales* order, *Negativicutes* class, *Firmicutes* phylum). Based on the distinctive physiological and phylogenetic characteristics of strain ALE^T^, a new genus and species *Lucifera butyrica* gen. nov., sp. nov., is proposed. The type strain is ALE^T^ (=JCM 19373^T^ = DSM 27520^T^). Strain ALE^T^ is an incomplete oxidizer and acetate, among other products, accumulates during glycerol conversion. Strain ALE^T^ was used to extend the substrate range for sulfur reduction by constructing co-cultures with the acetate oxidizer and sulfur reducer *Desulfurella amilsii.* The co-culture was tested with glycerol as substrate in batch and chemostat experiments. Acetate formed by fermentation of glycerol by strain ALE^T^ resulted in sulfur reduction by *D. amilsii*. The co-culture strategy offers good perspectives to use a wide range of cost-efficient substrates, including glycerol, to produce sulfide by specialized sulfur reducers. The recovery of heavy metals from metalliferous streams may become economically feasible by this approach.

**Note:** The locus tag for the genes encoded in *Lucifera butyrica* is LUCI_^∗^. To avoid repetition of the prefix along the text, the locus tags are represented by the specific identifier.

## Introduction

Respiration of sulfur compounds for the production of hydrogen sulfide is applied for the biotechnological recovery of metals (Sánchez-Andrea et al., 2014; Florentino et al., 2016b). Sulfate is naturally abundant in metalliferous streams such as acid mine drainage waters and, for this reason, most studies focus on its application as electron acceptor (Sánchez-Andrea et al., 2014; Atashgahi et al., 2018). However, the utilization of elemental sulfur has gained attention as, compared to sulfate, four times less electron donor is necessary for the production of an equivalent amount of sulfide (Florentino et al., 2015). The ability to reduce elemental sulfur is widespread among bacteria and archaea (Florentino et al., 2015) and sulfur-reducers can use a variety of electron donors, including hydrogen, alcohols, organic acids, sugars, starch and molasses (Bonch-Osmolovskaya et al., 1990; Finster et al., 1997; Dirmeier et al., 1998; Boyd et al., 2007). Recently, we described *Desulfurella amilsii*, a moderately acidophilic sulfur reducer with the ability to oxidize acetate to CO_2_. Substrate range of *D. amilsii* is rather limited and it is not capable of using, for instance, poly- and monosaccharides, alcohols or glycerol (Florentino et al., 2016a).

The cost of the electron donor is an important aspect in the application of microbial metal recovery by precipitation with sulfide. An attractive option is glycerol, an abundant by-product (10% w/w) of biodiesel production. Glycerol has been used as electron donor to enrich for acidophilic sulfate reducers and to stimulate dechlorination (Nancucheo and Johnson, 2012; Sánchez-Andrea et al., 2013; Atashgahi et al., 2018; Nino de Guzman et al., 2018). A few bacteria have been reported that are able to reduce sulfur while utilizing glycerol as energy and carbon source, such as *Desulfosporosinus meridiei* (Robertson et al., 2001), *Desulfosporosinus auripigmenti* (Stackebrandt et al., 2003), *Desulfosporosinus burensis* (Mayeux et al., 2013), *Desulfosporosinus acidiphilus* (Alazard et al., 2010), *Desulfovibrio cavernae* (Sass and Cypionka, 2004), and *Desulfovibrio indonesiensis* (Feio et al., 1998) but their performance at low pH might be hampered by acetic acid accumulation and inhibition.

In this work we aimed to broaden the range of substrates for acidophilic sulfur reduction by the co-cultivation of a specialized sulfur reducer, such as *D. amilsii*, with a versatile fermentative microorganism (strain ALE^T^). Strain ALE^T^ was isolated from acidic sediments of Tinto River (Spain) (Sánchez-Andrea et al., 2013) and here characterized. Among a large variety of substrates, the isolate was able to ferment glycerol to acetate and 1,3-propanediol (1,3-PDO) as main products. Here, we present a physiological and genetic characterization of strain ALE^T^, which is considered a novel species of a novel genus, for which the name *Lucifera butyrica* gen. nov., sp. nov. is proposed. The combined growth of strain ALE^T^ with the acidotolerant sulfur respirer and acetate oxidizer *D. amilsii* depleted the acetate produced during glycerol degradation by strain ALE^T^ and boosted sulfidogenesis by *D. amilsii*.

## Materials and Methods

### Source of the Organisms

A set of enrichment cultures targeting acidophilic sulfate-reducing bacteria was performed (Sánchez-Andrea et al., 2013) using as inoculum acidic sediments from Tinto River basin (southwestern Spain): *JL* dam (37.691207N, 6.560587W). Detailed information about the physicochemical characteristics of the sediment was previously described (Sánchez-Andrea et al., 2011). In that study, one of the enrichments showed growth, but no sulfate reduction. Clone library analyses indicated that the enriched bacterium was distantly related to *Propionispora hippei* (91.4% 16S rRNA gene sequence identity), an anaerobic propionate-producing fermenter. Strain ALE^T^ was subsequently isolated by performing three times serial dilution in liquid media, followed by streaking on agar plates (0.8% Agar noble, Difco) for three times and a last serial dilution. *P. hippei* (DSM 15287^T^) and *Propionispora vibrioides* (DSM 13305^T^) were purchased from the German Collection of Microorganisms and Cell Cultures (DSMZ) (Braunschweig, Germany). *D. amilsii* (DSM 29984^T^) was isolated and described previously in our laboratory (Florentino et al., 2016a) using the same inoculum as source of microorganisms and was taken from the internal culture collection of the Laboratory of Microbiology (Wageningen University, Netherlands).

### Media Preparation

An O_2_-free basal medium was prepared as previously described Stams et al. (1993), supplemented with 0.1 g l^-1^ yeast extract and replacing sulfide by 0.5 g l^-1^ L-cysteine sodium salt as reducing agent. Bicarbonate-buffer was also removed to allow pH modification. The gas phase of the cultures was set to 1.5 atm of N_2_/CO_2_ (80:20, v/v). Media was adjusted with HCl and NaOH to the different experimental pH values before autoclaving depending on the final desired pH value. All solutions were heat-sterilized, except for the vitamins which were filter-sterilized. Substrates were prepared in stocks of 1 M and added to the media at final concentrations ranging from 5 to 10 mM.

### Genome Analysis of Strain ALE^T^

Total genomic DNA of strain ALE^T^ was extracted with the MasterPure^TM^ Gram Positive DNA Purification Kit (Epicentre, Madison, WI, United States). DNA was sequenced at GATC Biotech (Konstanz, Germany) on an Illumina MiSeq Personal Sequencer, generating 887692 paired end reads with a length of 250 bp. Genome size was estimated by using k-mer spectrum analysis on the complete left end set of the paired-end reads. Assembly, merging and scaffolding of genome sequences were performed as described previously in Florentino et al. (2017). Assembled genome was manually filtered for 20 bp contigs. Automated annotation was performed using the Galaxy annotation server (Afgan et al., 2016), followed by manual curation. Additional gene prediction analysis and functional annotation were done within the Rapid Annotation using Subsystem Technology – RAST server (Aziz et al., 2008; Overbeek et al., 2014), and the Semantic Annotation Platform with Provenance – SAPP (Koehorst et al., 2018). RNA genes were *in silico* predicted using RNAmmer 1.2 (Lagesen et al., 2007). The draft genome sequence of strain ALE^T^ has been deposited at the European Nucleotide Archive under the accession numbers UPPP01000001-UPPP01000134.

### Phylogeny

The 16S rRNA gene sequence of strain ALE^T^ was aligned with SILVA Incremental Aligner (SINA) (Pruesse et al., 2012). The aligned sequence was merged with ARB program (Ludwig et al., 2004) in a database (Ref NR 99, released 13.12.2017) of over 695000 homologous prokaryotic 16S rRNA gene primary structures, and added by parsimony to the tree generated in the Living Tree Project (Yarza et al., 2008). Phylogenetic reconstruction was performed by using the maximum likelihood (RaxML), neighbor-joining (with Jukes-Cantor correction) and maximum parsimony algorithms as implemented in the ARB package. A consensus tree was generated with ARB v6.0 software. Pairwise comparison was performed taking into consideration the secondary structure of the 16S rRNA gene with the distance matrix method (similarity filter) implemented in ARB. The 16S rRNA gene sequence has been deposited in the EMBL database under accession number HG317005 and refers to the type strain ALE^T^.

Whole-genome phylogenetic reconstruction for 16 genomes belonging to *Sporomusaceae* and *Veillonellaceae* families (and *D. acidiphilus* as outgroup) was conducted with Prokka v1.13.3 (Seemann, 2014) with default parameters. The GFF3 files generated by Prokka were used for the core genes definition by Roary v3.12.0 (Page et al., 2015) with a setting of 80% for the minimum identity of BLASTp and maximum number of clusters 60,000. The core gene output of Roary was analyzed by RAxML using default parameters and generating a Newick tree with bootstrap values (Stamatakis, 2014). The iTOL tool (Letunic and Bork, 2006) was used for visualizing the phylogenetic tree.

### Phenotypic Characterization

Cell morphology, motility and spore formation of strain ALE^T^ were examined by phase contrast microscopy using a Leica DM2000 microscope (Leica Microsystems, Wetzlar, Germany). Scanning electron microscopy (SEM) was performed as previously described (Alphenaar et al., 1994) using a JEOL JSM-6480LV microscope (JEOL, Tokyo, Japan), the length and width of cells were measured, and mean dimensions recorded. Gram staining was performed according to standard procedures (Doetsch, 1981). Gram-structure was confirmed by checking the reaction of cells with 3% (w/v) solution of KOH. Catalase activity was determined by reaction with 3% (w/v) solution of H_2_O_2_. Oxidase test was performed with a filter impregnated in 1% (w/v) solution of tetramethyl-*p*-phenylenediamine in dimethyl sulfoxide (Sigma-Aldrich, St. Louis, MO, United States). Urease formation, as well as gelatin and esculin hydrolysis were determined with API^®^20A (bioMérieux, France), according manufacturer’s instructions.

Growth experiments were performed in triplicates, using 120 mL-serum bottles filled with 50 mL of anoxic media. Unless mentioned otherwise, physiological tests were done at pH 5.5, 37°C, and 5 mM of glycerol. Growth of strain ALE^T^ was studied in a range of temperature from 15 to 45°C, pH from 2.5 to 7.5 (in 0.5 pH intervals) and NaCl concentrations from 0.3 to 3.8% (w/v) in 0.5% intervals. Substrates were tested at a final concentration of 5 mM each or 10 g L^-1^ for starch, peptone, glycogen or yeast extract. Dependence on the aforementioned added vitamins and yeast extract was studied by removing them from the medium. Sensitivity of strain ALE^T^ to vancomycin, streptomycin, rifampicin, penicillin and chloramphenicol was determined at final concentrations of 25, 50, and 100 μg mL^-1^. Strain ALE^T^ tolerance to metals in solution was assessed at pH 3.5 (to enhance their solubility) with copper and zinc chloride salts, and iron and nickel sulfate salts targeting final concentrations of 1, 5, and 10 mM for copper and zinc; and 10, 20, and 50 mM for iron and nickel. To avoid precipitation of metals as metal sulfides, titanium citrate was used as reducing agent. To account for metal precipitation due to phosphate present in the medium, the real concentration of free metals available to the cultures at the beginning of the cultivation was first measured after their addition to the medium.

Soluble substrates and intermediates (sugars and volatile fatty acids) were measured using a Thermo Electron spectra system HPLC equipped with an Agilent Metacarb 67H column. Gaseous compounds (H_2_) were analyzed using a Shimadzu GC-2014 Gas Chromatograph equipped with a Molsieve 13X column. Sulfide was measured photometrically by the methylene blue method (Cline, 1969). Strain ALE^T^ tends to aggregate, therefore growth and generation times of cultures were determined from semi-logarithmic plots of changes in glycerol consumption or sulfide production values against time.

Membrane lipids fatty acids and quinones analyses were carried out at DSMZ (Braunschweig, Germany), with biomass grown on 5 mM of glycerol. For membrane lipids fatty acids comparison, *P. hippei* and *P. vibrioides* were also grown in the same anoxic basal medium on 5 mM glycerol.

### Co-culture Experiments

A monoculture of strain ALE^T^ and a combined culture with *D. amilsii* were analyzed in parallel and in triplicates, growing on 5 mM of glycerol and 25 mM of elemental sulfur. *D. amilsii* inoculum originated from a culture growing on acetate as electron donor and elemental sulfur as electron acceptor. Experiments were performed in 250 mL bottles with 100 mL of liquid phase, in which activity was monitored by sulfide production measurements. In order to be able to control the pH of the process, strain ALE^T^ monoculture and co-culture with *D. amilsii* were transferred from actively growing cultures in exponential phase to pH-controlled glass batch reactors of 1 L working volume (Applikon, Schiedam, Netherlands). The reactors operation was controlled by an ADI 1010 Bio-Controller with an ADI 1025 Bio-console (Applikon, Schiedam, Netherlands). The culture had a stirring speed of 25 rpm, temperature was controlled at 37°C, and the pH was maintained at 6.0 by automatic addition of 0.1 M of KOH or HCl. Growth and activity were weekly monitored off-line by counting the number of cells with a Petroff-Hausser counting chamber with a cell-depth of 0.02 mm and ruling pattern 1:400 mm2 (Hausser Scientific, Horsham, PA, United States), and measuring substrate and product profiles as aforementioned.

## Results and Discussion

### Characterization of Strain ALE^T^

Analysis of the 16S rRNA gene sequences of the isolated strain ALE revealed *P. hippei* with 91.4% 16S rRNA gene identity as the closest related species, followed by *P. vibrioides*, *Dendrosporobacter quercicolus*, and *Sporomusa* spp. (Supplementary Table S1). These species belong to the newly reclassified *Sporomusaceae* family –previously designated as *Selemonodales incertae sedis* 2- within the *Firmicutes* phylum (Campbell et al., 2015). Pairwise comparison analysis of the 16S rRNA sequence of strain ALE^T^ with members of *Sporomusaceae* family showed similarities within 86.5 and 94.5% -as thresholds for members of the same family but different genus (Yarza et al., 2014)- and less than 86.5% identity with members of *Veillonellaceae* family (Supplementary Table S1), indicating that strain ALE^T^ would represent a new genus of the *Sporomusaceae* family.

**FIGURE 1:**
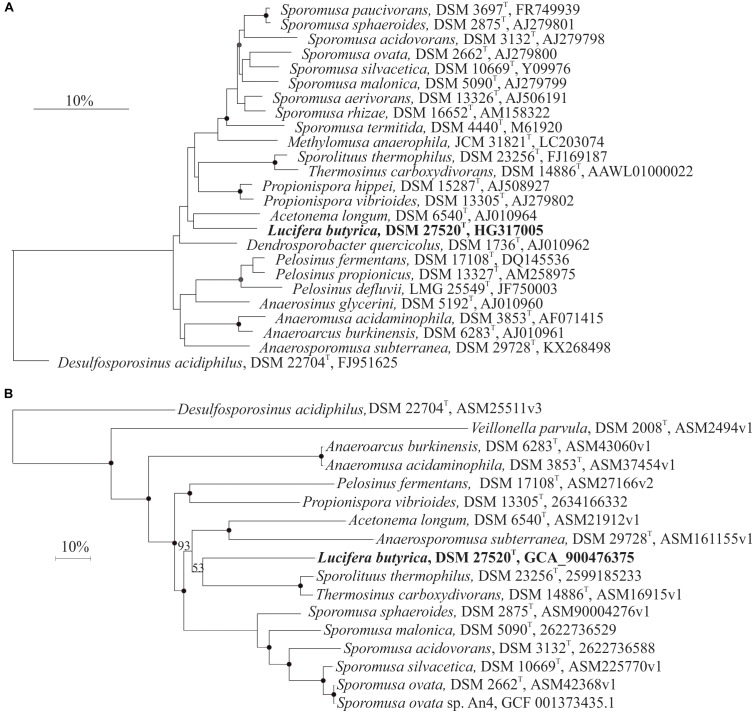
**(A)** 16S rRNA gene sequence phylogenetic reconstruction of strain ALE^T^ (bold type) and of related species in the *Sporomusaceae* family of the *Firmicutes* phylum. Consensus tree was performed after applying the maximum likelihood, neighbor-joining and maximum parsimony algorithms implemented in the ARB package. Trees were constructed with different filters (termini, subact, none) and based on 1000 replications. Bootstrap values greater than 70% are indicated by filled circles, and greater than 50% by gray circles. Bar indicates a 10% estimated sequence divergence. The sequence of *Desulfosporosinus acidiphilus* was used as outgroup. **(B)** Phylogenetic reconstruction with maximum-likehood of the core genes from 28 sequenced species closely related to *Lucifera butyrica* strain ALE^T^. Bootstrap of 100% are indicated by filled circles, and others by their number for clarity. Bar indicates a 10% estimated sequence divergence. The sequence of *Desulfosporosinus acidiphilus* was used as outgroup.

Campbell et al. (2015) showed that the phylogenetic reconstruction based on 16S rRNA gene analysis of the former *Selemonadales incertae sedis* presents limited resolution, observation confirmed in our 16S rRNA gene phylogenetic reconstruction of the *Sporomusaceae* family (Figure 1A). To support 16S rRNA analysis, we performed a phylogenetic reconstruction based on the core genes of the genomes of strain ALE^T^ and the 18 closest species with sequenced genomes (Figure 1B). This whole genome reconstruction show a stable separation within the different members of *Sporomusaceae* family and *Veillonella parvula* as representative of *Veillonellaceae* family with a bootstrap value of 100%. This confirms the placement of strain ALE^T^ in the *Sporomusaceae* family.

**FIGURE 2:**
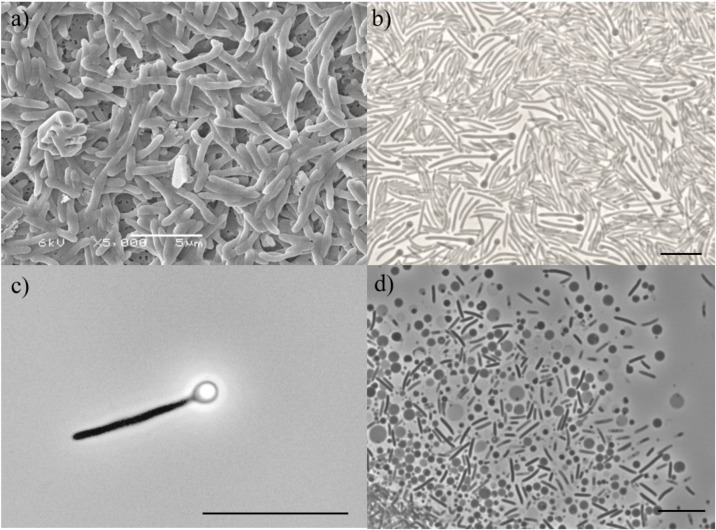
**(a)** Scanning electron microscopy image of cells of strain ALE^T^; Bar represents 5 μm. Phase-contrast microphotographs of **(b)** vegetative, **(c)** spore-forming cell with refractive sporangia in terminal position, and **(d)** vesicles of strain ALE^T^. Bar represents 2 μm.

Cells of strain ALE^T^ were rods, 4 – 5 μm in length and 0.6 μm in width (Figures 2a,b), occurring singly and showing motility during the exponential phase. Spores were readily formed in the growth conditions tested (Figure 2c). Vesicles were observed in stationary phase cultures (Figure 2d). Cells stained Gram-positive and the addition of KOH did not disrupt their cell-wall structure which confirmed the Gram-positive cell membrane structure. Strain ALE was strictly anaerobic; L-cysteine, ferrous iron or sulfide were required as reducing agents. The isolate tolerated up to 8 g L^-1^ of NaCl. Growth was observed at temperatures ranging from 20 to 40°C, with an optimum at 37°C, and at a pH range from 3.5 to 7.0, with optimum at 5.5. Growth by fermentation of glucose was also seen at pH as low as 3.0. The specific growth rate on glycerol under optimal growth conditions was 0.033 h^-1^ (generation time of 21 h). The dependency of growth rate on temperature or pH is given in Supplementary Figure S1.

Strain ALE^T^ was able to grow by fermentation of sugars, such as glucose, mannose, rhamnose, and xylose, organic acids such as succinate, citrate, malate and pyruvate, amino acids like serine, and glycerol. Complex compounds like yeast extract and peptone were also degraded by the isolate (Table 1). Typical products of the fermentation of sugars were hydrogen, acetate, butyrate and ethanol, which differs from the closely related *P. hippei* which mainly form acetate and propionate (Abou-Zeid et al., 2004). Fermentation of glycerol resulted also in the formation of 1,3-PDO. Measurements of substrates consumed, and products formed during fermentation and respiration processes are given in Supplementary Tables S2, S3, respectively.

**Table 1:** Differential characteristics between strain ALE^T^ and its closest relatives of the *Sporomusaceae* family, selected combining 16S rRNA gene similarity, and phylogenetic reconstructions based on 16S rRNA gene and core genes in the genome.

Characteristics	*Lucifera butyrica*	*Propionispora vibrioides^a^*	*Sporomusa sphaeroides^c^*	*Acetonema longum^d^*	*Pelosinus fermentans^e^*	*Dendrosporobacter quercicolus^g^*	*Thermosinus carboxydovorans^j^*
							
Strain	ALE^T^	FKBS^T^	E^T^	APO-1^T^	R7^T^	DSM 1736^T^	Nor1^T^
Temperature range (°C)	25–40	30–40	15–45	19–40	Oct-42	20–45	40–68
pH range	(3) 3.5–7.0	5.0–8.5	5.7–8.7	6.4–8.6	5.5–8.0	NT	6.5–7.6
Optimal pH	5.5	7.5	6.4–7.6	7.8	7.0–7.5	NT	6.8–7.0
Gram staining	Positive	Negative	Negative	Negative	Negative	Negative	Negative
DNA G+C content (mol%)	46.9	48.5	46.7–47.4	51.5	39.4	52–54	51.7

**Substrates**							

Arabinose	NT	NT	NT	-	NT	NT	+
Fructose	-	+	-	+	+	NT	+
Glucose	+	-	-	+	+	NT	+
Lactose	-	NT	-	-	NT	NT	+
Levulose	NT	NT	-	NT	NT	+	NT
Maltose	NT	NT	-	-	+	NT	+
Mannose	+	NT	-	+	-	NT	NT
Rhamnose	+	NT	-	+	-	NT	NT
Ribose	-	NT	-	+	NT	+	NT
Sucrose	-	NT	-	-	NT	NT	+
Xylose	+	-	-	-	-	NT	+
3-hydroxybutyrate	NT	NT	+	NT	NT	NT	NT
Aspartate	-	NT	-	NT	NT	NT	NT
Citrate	+	NT	-	+	+	NT	-
DL-glycerate	NT	NT	+	NT	NT	NT	NT
Glutamate	-	NT	-	NT	NT	NT	NT
Lactate	-	-	+	-	+	NT	-
Malate	+	NT	-	-	+	NT	NT
Pyruvate	+	-	+	+	+	NT	+
Succinate	+	NT	-	-	+	NT	NT
Glycerol	+	+	+	-	-	+	NT
Methanol	-	-	+	-	+	NT	NT
Betaine	NT	NT	+	NT	NT	NT	NT
*N,N*-dimethylethanolamine	NT	NT	+	NT	NT	NT	NT
*N,N*-dimethylglycine	NT	NT	+	NT	NT	NT	NT
Trimethylamine	NT	NT	+	NT	NT	NT	NT

**Electron Acceptors**							

DMSO	+	-	NT	NT	NT	NT	NT
Ferric Iron	+	NT	NT	NT	+	NT	+

**Main products of sugar fermentation**							

Propionate	-	+	-	+	+	NT	-
Butyrate	+	-	-	+	-	NT	-
Ethanol	+	-	+	-	-	NT	-
Acetate	+	+	+	+	+	NT	+
Succinate	-	-	-	-	-	NT	-
Propanol	-	-	-	-	-	NT	-


*+, Positive; -, Negative, NT, not tested. All strains needed yeast extract, were spore-formers, and could utilize acetate. Optimum temperature for all the strains is 37°C. ^a^Biebl et al. (2000), ^b^Möller et al. (1984), ^c^Kane and Breznak (1991), ^d^Shelobolina et al. (2007), ^e^Stankewich et al. (1971), ^f^Sokolova et al. (2004).*

The strain was able to use thiosulfate, DMSO and iron as electron acceptors. We also tested its ability to reduce elemental sulfur. As L-cysteine was used as reducing agent in the medium, controls were performed with L-cysteine and glycerol. These cultures produced 0.75 mM of sulfide and when sulfur was added, 1.24 mM of sulfide was formed. L-cysteine is the main source of sulfide formation, but cysteine alone did not support growth or sulfidogenic activity.

Strain ALE^T^ was able to grow in the presence of vancomycin and streptomycin at concentrations up to 100 and 25 μg ml^-1^, respectively. No growth was observed at any tested concentration of chloramphenicol, penicillin, or rifampicin. At pH 3.5, the isolate was able to grow with up to 1 mM of zinc and 50 mM of iron in solution. Major components in the fatty acid profile of strain ALE^T^ were palmitic acid - C_16:0_ (22.66%) and palmitoleic acids - C_16:1_ w9c (13.77%) and C_16:1_ w7c (13.01%). Cellular fatty acid composition profiles of strain ALE^T^ differed significantly from its closest phylogenetic relatives (Supplementary Table S4). The two closest *Propionispira* species formed C_11:0_, C_15:0_, C_17:0_, and anteiso-C_15:0_ in high abundance. The only quinone component detected was menaquinone MK6.

Strain ALE^T^ shared a fermentative metabolism with its closest relatives of the *Sporomusaceae* family but, as expected due to its isolation source, it showed a high tolerance at low pH and certain metal tolerance. Their differential characteristics are shown in Table 1.

Based on the distinctive ecological, physiological and chemotaxonomical characteristics of strain ALE^T^, a new genus and species *Lucifera butyrica* gen. nov., sp. nov., is proposed. The type strain is ALE^T^ ( = JCM 19373^T^ = DSM 27520^T^).

#### Glycerol Fermentation

**FIGURE 3:**
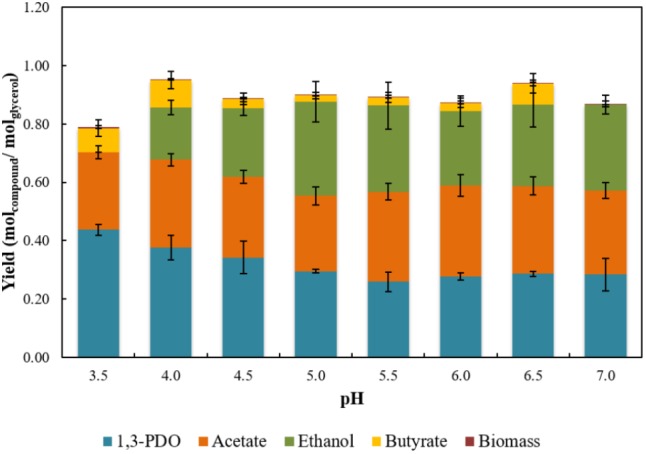
Yields of 1,3-PDO, acetate, ethanol, and butyrate achieved in stationary phase from glycerol fermentation by strain ALE^T^ in a range of pH from 3.5 to 7.0. Results are averaged from triplicates and the standard deviation values are displayed in brackets.

Strain ALE^T^ was shown to produce 1,3-PDO together with acetate, ethanol and butyrate during glycerol fermentation (Supplementary Table S2). The three-carbon diol is an important monomer in the synthesis of polyesters for fabric and textile applications (Saxena et al., 2009), and its biological production is of great interest due to the low selectivity and low production of toxic intermediates (Tang et al., 2009). In Figure 3, the effect of initial pH on glycerol fermentation is presented. The 1,3-PDO production per mol of glycerol consumed varied from 0.26 to 0.43, with an efficiency of glycerol degradation from 79.4 to 84.7%. Cultures incubated at pH 3.5 and 4.0 had the highest 1,3-PDO yields, 0.43 and 0.40 mol mol^-1^_glycerol_, respectively. At optimal pH for growth (pH 5.5) of the isolate, ethanol and acetate represented circa of 60% of the products, while 1,3-PDO had its lowest yield, 0.26 mol mol^-1^_glycerol._ However, acetate production per mol of glycerol varied from 0.26 to 0.33, the highest acetate production was at pH 6, therefore this pH value was selected for the reactor operation in order to maximize the substrate provided to *D. amilsii.*

### Co-culture Experiments

Apart from accumulation of products, such as acetate, poor sulfidogenesis was observed when strain ALE^T^ grew in the presence of glycerol and elemental sulfur in a cysteine-containing medium. Therefore, co-cultivation experiments of strain ALE^T^ together with the acetate oxidizer *D. amilsii* were performed to assess the possible boost in sulfide production coupled to glycerol degradation. Strain ALE^T^ showed a versatile fermentative metabolism using a variety of substrates that cannot be used by *D. amilsii*, a sulfur reducer isolated from the same acidic environment, Tinto river. We hypothesized that strain ALE^T^ and *D. amilsii* can complement each other. In our co-culture experiment, glycerol was mainly fermented by strain ALE^T^, and the acetate produced from glycerol was used by *D. amilsii* as substrate for sulfur reduction.

**FIGURE 4:**
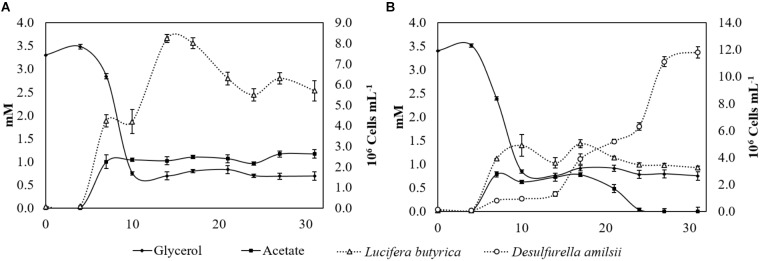
**(A)** Glycerol consumption, acetate production and number of cells of strain ALE^T^ growing in a pH-controlled batch reactor with glycerol and sulfur. 1,3-PDO (1.07 mM), ethanol (0.72 mM), butyrate (0.26 mM), and propionate (0.42 mM) production was not displayed in the graph for clarity purposes. **(B)** Glycerol, acetate and number of cells profile of strain ALE^T^ growing in co-culture with *D. amilsii* in a pH-controlled batch reactor with glycerol and sulfur. 1,3-PDO (1.33 mM), ethanol (0.19 mM), butyrate (0.15 mM), and propionate (0.35 mM) production was not displayed in the graph for clarity purposes.

Strain ALE^T^ grown in a sulfur-added and pH-controlled chemostat (pH 6) with consumption of 3.5 mM glycerol formed 0.95 mM 1,3-PDO, 1.47 mM acetate, 0.55 mM ethanol, 0.35 mM butyrate and 0.53 mM propionate. The formation of propionate was only observed in the pH-controlled chemostat, and not in batch bottles cultivation. Moreover, the amount of ethanol produced in the first 17 days of chemostat cultivation reached 1.6 mM, decreasing to 0.55 mM after 31 days. In co-culture, 1.21 mM 1,3-PDO, 0.28 mM ethanol, 0.21 mM butyrate, and 0.42 mM propionate were measured. Additionally, up to 0.5 mM ethanol was produced in 17 days and this decreased to 0.2 mM after 31 days of cultivation. The consumption of glycerol by strain ALE^T^ alone or in co-culture ceased between day 10 and day 15 of cultivation, with circa of 0.7 mM of glycerol remaining in the medium. Growth of strain ALE^T^ in both culture conditions consequently stopped when glycerol consumption did (Figures 4A,B). The concentration of acetate in the pure culture stagnated after 7 days, when it reached its maximum concentration of 1.1 mM (Figure 4A). In the co-culture, acetate started to be produced after day 5, reaching its maximal detected amount (0.79 mM), and after 24 days of cultivation it was completely depleted. *D. amilsii* growth in co-culture neatly followed the presence of acetate: it started after 5 days of cultivation, when acetate was available in the culture, and it ceased when acetate was depleted (Figure 4B). In the pure culture of strain ALE^T^ about 1.05 mM sulfide was produced, likely from cysteine cleavage, while in the co-culture with *D. amilsii* about 10.9 mM sulfide was formed.

Sulfidogenesis based on microbial elemental sulfur respiration has been reported as an attractive alternative for metals sulfide precipitation (Florentino et al., 2015, 2016b). Many sulfidogenic microorganisms are incomplete oxidizers, contributing to the accumulation of acetic acid in the system, which might commonly lead to the inhibition of the process (Widdel and Pfennig, 1991; Castro et al., 2002; Sánchez-Andrea et al., 2015; Florentino et al., 2016b). Sulfur reducers able to completely oxidize and/or to directly utilize acetate to produce sulfide are therefore of great interest for metals precipitation processes. In this study, we show that coupling sulfide production to the degradation of low-cost substrates is an attractive strategy to the current used sulfidogenic systems. Besides, this strategy opens up the range of research in sulfur reducers growing by co-cultivation with fermenting-microorganisms, which might have their activity boosted via mechanisms likely not yet reported.

### Further Exploration of *L. butyrica* Strain ALE^T^ on Genome Basis

#### General Characteristics

Manually curated and annotated draft genome sequence of strain ALE^T^ comprises a chromosome with the size of 4.67 Mbp distributed over 138 scaffolds. The total coverage over the predicted genome size was 90% and the G+C content 46.96 mol%. A total of 5223 genes are predicted, from which 84 are tRNA and 12 are rRNA genes, with a single copy of 16S, a single copy of 23S and 6 copies of 5S rRNA genes. From the 5122 coding DNA sequences (CDS), 4158 have function prediction and 964 could not be assigned to any function in the database, and therefore were annotated as hypothetical proteins or proteins of unknown function. One CRISPR region (Type I-B) was identified in the genome with a length of 945 bp and 14 spacers of 30 bp length.

The genome encodes an incomplete tricarboxylic acid (TCA) cycle pathway, as genes encoding succinyl coA synthetase and α-ketoglutarate dehydrogenase are absent. Routes for glycerol fermentation leading to 1,3-PDO, acetate, butyrate and ethanol, resistance to vancomycin, acidic conditions, oxygen stress, and metals are encoded as well as genes possibly involved in sulfur and thiosulfate reduction (Supplementary Table S5).

#### Sulfur and Energy Metabolism

Sulfide production was observed when strain ALE^T^ was grown in the presence of L-cysteine and sulfur. Therefore, a genome exploration was performed to reveal further aspects on the physiology of the novel isolate. Genes encoding rhodanese-like sulfurtransferases were detected in the genome of strain ALE^T^ (0570, 0603, 1577, 3290). The involvement of rhodanese-like sulfurtransferases in sulfur metabolism is not clearly understood, although it has been recently associated with sulfur respiration processes in *D. amilsii* (Florentino et al., 2018). Physiological tests on strain ALE^T^ revealed its ability to utilize thiosulfate as electron acceptor, producing up to 4.5 mM of sulfide and although genes for the enzyme thiosulfate reductase were not detected in the draft genome, the dissimilatory sulfite reductase genes (1126, 1128, 1129) are encoded in strain ALE^T^ genome, implying that sulfite might be an intermediate in thiosulfate reduction, as typically observed (Florentino et al., 2018). However, there are three thiosulfate sulfurtransferases encoded (3290, 0570, and 0603) which might be involved in the reduction of thiosulfate.

The genome encodes genes of the bifurcating enzyme ferredoxin:NADP oxidoreductase (NfnA - 1587, 1907, 2081, 2596, 2903, 2905, 3092, 3164, 3176, 4378). The transhydrogenase NfnA activity *in vivo* has been limited to the carbon source rather than to elemental sulfur, enabling high potential donors to reduce low potential ferredoxin (Ma and Adams, 2001). However, this enzyme was previously shown to have sulfur-reducing activity *in vitro*. It might be that strain ALE^T^ can reduce elemental sulfur to some extent, forming small amounts of sulfide (as the 1.24 mM observed), but that needs to be further investigated.

Cysteine desulfhydrase genes are not encoded in strain ALE^T^ genome. However, the sulfide generated from the presence of cysteine in strain ALE^T^ cultures might be explained by a cysteine transamination activity conferred by cysteine/aspartate aminotransferase (0413, 0613, 2048, 2196, 3978, 4902) and 3-mercaptopyruvate sulfurtransferase (0570) enzymes, for which genes are encoded in its genome. The first cysteine/aspartate aminotransferase is involved in the initial step in the transamination pathway for degradation of cysteine, catalyzing the conversion of L-cysteine and 2-oxoglutarate to 3-mercaptopyruvate and L-glutamate, as described previously (Andreessen et al., 2017). The enzyme 3-mercaptopyruvate sulfurtransferase then convert the 3-mercaptopyruvate formed to pyruvate and hydrogen sulfide, likely from its association with thioredoxin and dihydrolipoic acid, as shown by Mikami et al. (2011).

#### Glycerol Degradation Pathway

As an uncharged molecule, glycerol is able to cross the microbial membrane by passive diffusion. In strain ALE^T^, a 28 kDa integral membrane aquaglyceroporin, GlpF, is encoded (1689, 2547, and 0660), but no active uptake systems like the symporters Na^+^/glycerol and H^+^/glycerol. Therefore, glycerol might diffuse into the cytoplasm of this bacterium via the channel protein.

Glycerol is fermented via oxidative and reductive pathways (Zhu et al., 2002). The reductive pathway is catalyzed by coenzyme B_12_-dependent glycerol dehydratase (4400), converting glycerol to 3-hydroxypropionaldehyde, and by the NADH-dependent enzyme 1,3-PDO dehydrogenase (1837), which reduces 3-hydroxypropionaldehyde to 1,3-PDO regenerating NAD^+^. In the oxidative pathway, a phosphate is added to a molecule of glycerol by glycerol kinase (4985, 4986), forming glycerol 3-phosphate, that will be reversibly converted to dihydroxyacetone-P by a NAD^+^-dependent glycerol dehydrogenase (1377, 2837). The glycolytic enzyme triose phosphate isomerase (2987, 3004) can catalyze its reversible conversion to glyceraldehyde-3P, which follows the glycolysis pathway to the formation of pyruvate. Physiological tests showed further conversion of pyruvate in strain ALE^T^ leading to the formation of acetate, butyrate, ethanol, and CO_2_. The genomic set of this isolate encode genes for acetate formation via the enzymes pyruvate:ferredoxin oxidorreductase (3166, 3177, 4376, 1480, 2083, 2907, 1907), converting pyruvate into acetyl-CoA; phosphate acetyl transferase (1551, 1554, 164, 2904, 3827), converting acetyl-CoA into acetyl-P; and acetate kinase (0876), converting acetyl-P into acetate. The direct conversion of acetyl-CoA to acetate might also be possible, as strain ALE^T^ encodes the acetyl-CoA hydrolase (1562). When acetyl-CoA is formed, however, it can also go to the butyrate formation pathway by formation of acetoacetyl-CoA via an acetyl-coenzyme A acetyltransferase (2274) and further conversion to butyryl-CoA via the hydroxybutytyl-CoA dehydrogenase (2124, 2280). A phosphotransbutyrylase transfers butyryl-CoA to butyryl-phosphate, after which butyrate kinase transfers the phosphate onto ADP, creating ATP. Acetyl-CoA might also be an intermediate in the formation of ethanol, with acetaldehyde as an intermediate. The first conversion is mediated by aldehyde dehydrogenase (0513, 1408, 4384, 5024), while the second occurs via one alcohol dehydrogenase (0150, 0956, 1837, 2263, 2290, 2523, 2947, 3895, 4400, 4514, 4887).

**FIGURE 5:**
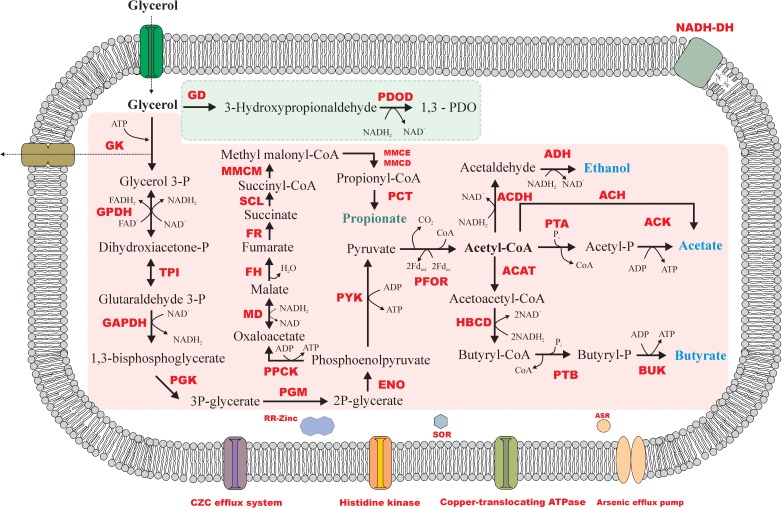
Metabolic reconstruction of glycerol fermentation by strain ALE^T^. Reductive and oxidative routes of glycerol degradation are represented by the green and the pink area, respectively. GD, glycerol dehydratase; PDOD, 1,3-PDO dehydrogenase; GK, glycerol kinase; GPDH, glycerol-3P dehydrogenase; TPI, triose phosphate isomerase; GAPDH, glyceraldehyde-3P dehydrogenase; PGK, phosphoglycerate kinase; PGM, phosphoglycerate mutase; ENO, enolase; PYK, pyruvate kinase; PFOR, pyruvate:ferredoxin oxidoreductase; PTA, phosphotransacetylase; ACK, acetate kinase; ACAT, acetyl-CoA acetyltransferase; HBCD, hydroxybutyryl dehydrogenase; PTB, phosphotransbutyrylase; BUK, butyrate kinase; ACDH, acetaldehyde dehydrogenase; AD, alcohol dehydrogenase; PPCK, phosphoenolpyruvate kinase; MD, malate dehydrogenase; FH, fumarate hydratase; FR, fumarate reductase; SCL, succinyl-CoA ligase; MMCM, methyl malonyl-CoA mutase; MMCE, methyl malonyl-CoA epimerase; MMCD, methyl malonyl-CoA dehydrogenase; PCT, propionyl-CoA transferase; RR-Zinc, response regulator of zinc; SOR, superoxide reductase; ASR, arsenate reductase. Propionate was only found in the pH controlled chemostat experiment.

Some studies reported butanol, 2,3-butanediol, lactate, succinate, 1,2-propanediol and propionate as products of microbial glycerol degradation (Ouattara et al., 1992; Biebl, 2001; Li et al., 2013). Although the genes encoding enzymes involved in the production of those compounds were present in the genome of strain ALE^T^, they were not detected in physiological tests performed in 120-mL serum bottles without pH control. However, in pH-controlled batch reactors some propionate was produced during glycerol degradation (described below). Figure 5 displays the glycerol degradation pathway likely performed by strain ALE^T^.

#### Antibiotic Resistance

Strain ALE^T^ was able to grow in the presence of up to 100 μg ml^-1^ of vancomycin, an antibiotic generally affecting bacteria with a Gram-positive cell membrane structure. The resistance to vancomycin results from the production of modified peptidoglycan precursors terminating in either D-alanyl-D-lactate (D-Ala-D-Lac) or D-alanyl-D-serine (D-Ala-D-Ser) to which vancomycin exhibits low binding affinities. The key enzyme for the production of the modified precursors is encoded by the D-alanyl-D-alanine ligase gene VanA and its homologues. Such enzyme is encoded in strain ALE^T^ (2001), as well as a two-component system regulator histidine kinase (VanR/VanS – 1643-1644), a uridine 5′-diphospho-*N*-acetylmuramoyl-tripeptide-D-alanyl-D-alanine ligase (MurF - 1439), a phospho-*N*-acetylmuramoyl-pentapeptide-transferase (MraY – 1438), and a uridine 5′-diphospho-*N*-acetylglucosamine-*N*-acetylmuramyl-pentapeptide (MurG – 1030).

#### Acid Resistance

Strain ALE^T^ was isolated from sediments of the extreme acidic Tinto river. Microorganisms living in these environments can develop various effective mechanisms to thrive at acidic conditions (Baker-Austin and Dopson, 2007). Strain ALE^T^ was shown to grow at pH from 3 (with glucose) or 3.5 (with glycerol) to 7.0 with an optimum at pH 5.5. Genes possibly involved in strain ALE^T^ resistance to low pH were investigated (Supplementary Table S5).

The encoded pH-dependent amino acid (0713, 2372, 2664) and ABC phosphate transporters (1612) are related to acid resistance by utilizing ATP energy bond and hydrolysis to transport ions and amino acids across cellular membranes (Kanjee and Houry, 2013). Moreover, potassium-transporting ATPase (2313, 2314, 2315) and the regulating histidine kinase (2311, 2312) have been related to the creation of a Donnan potential to compensate for the high proton motive force in low pH environments (Mirete et al., 2017). A consequence of the high redox potential present of the AMD environment can be the exposure to several reactive oxygen species (ROS). Glutaredoxin (1706, 4105) and rubredoxin (1096, 5138, 3460) are encoded in the genome, reported to be involved in the reduction of these ROS.

The genome encodes a DNA repair system that includes RecA (1219), MutS (1208, 4052), and a RecA regulator (RecX - 1218). RecA is reported to play a central role in biological processes that require homologous DNA repair and recombination, and SOS response, a global response to DNA damage (Adikesavan et al., 2011). Moreover, a RecA-dependent acid-tolerance system has also been reported for *Helicobacter pylori*. Strains lacking the RecA gene showed sensitivity to DNA-damaging agents and a reduction in conversion of homologous gene related to outer membrane protein expression, resulting in a reduced survival capacity in acidic environments (Thompson and Blaser, 1995; Amundsen et al., 2008). The DNA repair system UvrABC is also encoded in the genome of strain ALE^T^ (0694). Although there is no evidence of correlation between RecA system genes and microorganism’s survival at low pH, such system is encoded in several acidophilic/acidotolerant strains, such as *D. amilsii* (Florentino et al., 2017), *Thiobacillus ferrooxidans* (Valdés et al., 2008) and *Ferroplasma acidiphilum* (Golyshina et al., 2017). Furthermore, the genome of strain ALE^T^ also encodes one oxalate:formate antiporter (3096), reported by microarray experiments to be upregulated in cells of *Escherichia coli* when they undergo cytoplasmic acidification by treatment with benzoate (Kannan et al., 2008).

#### Tolerance to Heavy Metals in Solution

Strain ALE^T^ was able to grow with up to 1 mM of zinc and 50 mM of iron in solution. Several genes encoding resistance to both metals are encoded in the genome of strain ALE^T^, such as the cobalt-zinc-cadmium resistance genes (1692, 2763, 2963, 2975, 1700, 2101), their transcriptional regulator (0108, 0153, 0163, 2633, 3905, 4895, 4973, 0101) and the response regulator of zinc sigma-54-dependent two-component system (1031, 1930, 1938, 2953, 2955, 4071, 4072, 4079, 4080, 4146, 4421, 5023). Although strain ALE^T^ was sensitive to copper even at low concentration, its genome encodes genes for copper resistance, such as the multicopper oxidase (4187) (Djoko et al., 2008) and the copper-translocating P-type ATPase (3071, 3935). A ZIP zinc transporter (4845), related with the export of metals from the cytoplasm, is also encoded (Ma et al., 2009).

Additionally, genes for arsenic resistance are encoded, such as the arsenical resistance operon repressor (4106), arsenical resistance operon trans-acting repressor (1662), arsenical pump-driving ATPase (1661, 2676), arsenic efflux pump protein (2859, 3114), arsenate reductase (1669, 1685, 4104, 4171, 4174) and arsenical-resistance protein ACR3 (1667, 1687, 4172). The two mentioned families of arsenite transport proteins responsible for As(III) extrusion, ArsABC and Acr3, have been shown to confer arsenic resistance to *Staphylococcus aureus*, *Bacillus subtilis* (Rosen, 2002), as well as to some soil bacteria (Achour et al., 2007). Arsenic is one of the most harmful elements for the environment and human beings, and together with iron, cadmium, copper and zinc, it is frequently associated with the oxidation of metallic sulfide ore bodies (Cruz-Hernández et al., 2017). The isolation source of strain ALE^T^ is an acid rock drainage environment, characterized by its low pH and high concentrations of heavy metals in solution (Sánchez-Andrea et al., 2011). Therefore, the metals resistance genes encoded in the isolate’s genome corroborates its exposition and survival to metals-rich environments.

### Description of *Lucifera* gen. nov.

Lucifera (Lu.ci’fe.ra. L. fem. adj. used as a fem. n. *Lucifera*, light-bringing because of the match shape).

Lu.ci’fe.ra. L. fem. n. *lux lucis* light; L. suff. -*fer* -*fera* -*ferum* (from L. v. *fero* to bear) bearing; N.L. fem. n. *Lucifera* light-bearing.

Cells are spore-forming, motile long rods that stain Gram-positive. Strictly anaerobic. Yeast extract is required for growth. Optimum growth temperature is 37°C within a range of 25–40°C. Optimum growth pH is 5.5 within a range from 3.5 to 7.0. Negative for both oxidase and catalase and positive for urease activity. Gelatin hydrolysis occurred, but aesculin hydrolysis not. Species has a fermentative metabolism using sugars, some organic acids, some aminoacids and glycerol. Major cellular fatty acids are C_16:0_, C_16:1_ w9c and C_16:1_ w7c. The only respiratory quinone detected was MK6. The genomic DNA G+C content of the type species is 47 mol%. The type species is *Lucifera butyrica*.

The genus *Lucifera* is classified within the family *Sporomusaceae*.

### Description of *Lucifera butyrica* sp. nov.

Lucifera butyrica (bu.ty’ri.ca. N.L. neut. n. *acidum butyricum* butyric acid; N.L. fem. adj. *butyrica* pertaining to butyric acid).

Morphology and general characteristics are as described for the genus. Cells are motile long rods, 0.4–0.6 μm in diameter and 5 μm in length. The temperature range for growth is 25–40°C, with an optimum at 37°C. The pH range for growth is 3.5–7.0, with an optimum at 5.5. NaCl was tolerated in concentrations up to 0.8% (w/v). It fermented sugars (glucose, mannose, rhamnose, and xylose), organic acids (succinate, pyruvate, malate, and citrate), glycerol and complex substrates such as yeast extract and peptone. Acetate, butyrate, ethanol, and hydrogen are formed from sugars. Has the ability to use thiosulfate, iron and DMSO as electron acceptors. Nitrate, arsenate, sulfate, sulfite, elemental sulfur, perchlorate, and fumarate are not used as electron acceptor. Oxidase and catalase activities were negative, urease was positive. Indole formation was negative. Gelatin, but not aesculin was hydrolysed. It tolerates up to 1 mM of zinc and 50 mM of iron in solution and is able to grow in the presence of up to 100 and 25 μg ml^-1^ of vancomycin and streptomycin, respectively. Chloramphenicol, penicillin and rifampicin do not allow growth. The predominant cellular fatty acids were C_16:0_, C_16:1_ ω9c and C_16:1_ ω7c. Menaquinone MK6 was the only respiratory quinone. The G+C content of the genomic DNA of the type strain is 46.96 mol%.

The type strain, ALE^T^ ( = JCM 19373^T^ = DSM 27520^T^), was isolated from sediments of an acid rock drainage environment (Tinto River, Spain).

## Conclusion

A novel fermentative bacterium, *Lucifera butyrica* strain ALE^T^, was isolated and described. The ability of the isolate to grow in a broad range of pH and tolerating high concentrations of zinc reflects the conditions of its natural habitat, where it lives together with acidophilic sulfate- and sulfur-reducing bacteria. The co-culture strategy is a good opportunity to combine an acetate-producing bacterium using a range of cost-efficient substrates, such as glycerol, with an acetate-oxidizing bacterium with good sulfur reduction abilities in order to precipitate heavy metals from metalliferous waste streams. Further studies at lower pH values and in a continuous mode system with addition of heavy metals are required to optimize the combined growth of the microorganisms, assuring metals precipitation. It needs to be studied further if the ability *L. butyrica* to form 1,3-PDO from glycerol is of interest for biotechnology.

## Data Availability Statement

The genomic dataset generated and analyzed for this study has been deposited at the European Nucleotide Archive under the accession numbers UPPP01000001-UPPP01000134.

## Author Contributions

IS-A isolated the strain. IS-A, AF, and JS performed the experiments in the lab. All authors analyzed the genome and critically reviewed the manuscript.

## Conflict of Interest Statement

The authors declare that the research was conducted in the absence of any commercial or financial relationships that could be construed as a potential conflict of interest.
