# Supplementary material for: Co-culture of a Novel Fermentative Bacterium, Lucifera butyrica gen. nov. sp. nov., With the Sulfur Reducer Desulfurella amilsii for Enhanced Sulfidogenesis
Source: Front Microbiol. 2018 Dec 13;9:3108. doi: 10.3389/fmicb.2018.03108 (PMC6315149; doi:10.3389/fmicb.2018.03108)
Supplement: Supplementary file 1 [file Table_1.DOCX]

Supplementary Material

Co-culture of a novel fermentative bacterium, *Lucifera butyrica* gen. nov. sp. nov., with the sulfur *Desulfurella amilsii* for enhanced sulfidogenesis

**Irene Sánchez-Andrea^1*^, Anna Patricya Florentino^1*^, Jeltzlin Semerel^1^*,* Nikolas Strepis^1,2^, Diana Z. Sousa^1^, and Alfons J.M. Stams^1,3^**

^1^Laboratory of Microbiology, Wageningen University, Stippeneng 4, 6708 WE, Wageningen, The Netherlands.

^2^Laboratory of Systems and Synthetic Biology, Wageningen University, Stippeneng 4, 6708 WE, Wageningen, The Netherlands.

^3^CEB-Centre of Biological Engineering, University of Minho, Campus de Gualtar, 4710-057, Braga, Portugal.

*both authors contributed equally.

**Correspondence:**Irene Sánchez Andrea
Irene.sanchezandrea@wur.nl

Keywords: Acidophilic sulfur reduction, glycerol, 1,3-PDO, *Lucifera* *butyrica*, *Desulfurella amilsii,* coculture.

**Supplementary Figure 1.** Effect of (a) temperature and (b) pH on growth rates of strain ALE^T^ (grown by fermentation of 5 mM glycerol at 37 °C).

**a**

**b**
