# Supplementary material for: Co-culture of a Novel Fermentative Bacterium, Lucifera butyrica gen. nov. sp. nov., With the Sulfur Reducer Desulfurella amilsii for Enhanced Sulfidogenesis
Source: Front Microbiol. 2018 Dec 13;9:3108. doi: 10.3389/fmicb.2018.03108 (PMC6315149; doi:10.3389/fmicb.2018.03108)
Supplement: Supplementary file 2 [file Table_2.DOCX]

Supplementary Material

Co-culture of a novel fermentative bacterium, *Lucifera butyrica* gen. nov. sp. nov., with the sulfur *Desulfurella amilsii* for enhanced sulfidogenesis

**Irene Sánchez-Andrea^1*^, Anna Patricya Florentino^1*^, Jeltzlin Semerel^1^*,* Nikolas Strepis^1,2^, Diana Z. Sousa^1^, and Alfons J.M. Stams^1,3^**

^1^Laboratory of Microbiology, Wageningen University, Stippeneng 4, 6708 WE, Wageningen, The Netherlands.

^2^Laboratory of Systems and Synthetic Biology, Wageningen University, Stippeneng 4, 6708 WE, Wageningen, The Netherlands.

^3^CEB-Centre of Biological Engineering, University of Minho, Campus de Gualtar, 4710-057, Braga, Portugal.

*both authors contributed equally.

**Correspondence:**Irene Sánchez Andrea
Irene.sanchezandrea@wur.nl

Keywords: Acidophilic sulfur reduction, glycerol, 1,3-PDO, *Lucifera* *butyrica*, *Desulfurella amilsii,* co-culture.

**Supplementary Table S1 –** Pairwise comparison of the 16S rRNA gene of *Lucifera butyrica* with the closest isolates. Cells highlighted in red correspond to the three type strains with highest similarity. Cells highlighted in yellow correspond to those species bellow the threshold of 86.5% (Yarza et al. 2012) of 16S rRNA gene for family level differentiation.

| Type strains, accession numbers |  |
| --- | --- |
| *Propionispora hippei*, AJ508927 | 91.4 |
| *Dendrosporobacter quercicolus*, AJ010962 | 91.3 |
| *Propionispora vibrioides*, AJ279802 | 91.0 |
| *Sporomusa sphaeroides*, AJ279801 | 90.8 |
| *Sporomusa paucivorans*, FR749939 | 90.4 |
| *Sporomusa acidovorans*, AJ279798 | 90.4 |
| *Sporomusa rhizae*, AM158322 | 90.3 |
| *Acetonema longum*, AJ010964 | 90.1 |
| *Sporomusa silvacetica*, Y09976 | 89.9 |
| *Sporomusa aerivorans*, AJ506191 | 89.9 |
| *Pelosinus propionicus*, AM258975 | 89.9 |
| *Sporomusa malonica*, AJ279799 | 89.5 |
| *Sporomusa ovata*, AJ279800 | 89.5 |
| *Sporomusa termitida*, M61920 | 89.4 |
| *Pelosinus fermentans* DSM 17108, DQ145536 | 89.4 |
| *Anaeroarcus burkinensis*, AJ010961 | 89.4 |
| *Sporolituus thermophilus*, FJ169187 | 89.2 |
| *Anaerosporomusa subterranea*, KX268498 | 89.0 |
| *Anaeromusa acidaminophila*, AF071415 | 88.8 |
| *Anaerospora hongkongensis*, AY372050 | 88.7 |
| *Thermosinus carboxydivorans* Nor1, AAWL01000022 | 88.7 |
| *Anaerosinus glycerini*, AJ010960 | 88.5 |
| *Pelosinus defluvii*, JF750003 | 87.4 |
| *Veillonella seminalis,* AY211542 | 86.5 |
| *Megasphaera cerevisiae*, L37040 | 85.6 |
| *Veillonella criceti*, AF186072 | 85.6 |
| *Veillonella magna*, EU096495 | 85.6 |
| *Veillonella rodentium*, AY514996 | 85.5 |
| *Veillonella tobetsuensis*, AB679109 | 85.3 |
| *Veillonella rogosae*, EF108443 | 85.2 |
| *Veillonella ratti*, AY355138 | 85.2 |
| *Megasphaera elsdenii*, U95027 | 85.1 |
| *Megasphaera paucivorans*, DQ223730 | 85.1 |
| *Megasphaera sueciensis*, DQ223729 | 85.1 |
| *Veillonella caviae*, AY355140 | 85.1 |
| *Veillonella atypica*, AF439641 | 85.1 |
| *Veillonella denticariosi*, EF185167 | 85.0 |
| *Megasphaera indica*, HM990965 | 84.9 |
| *Megasphaera micronuciformis*, AF473834 | 84.9 |
| *Dialister micraerophilus*, AF473837 | 84.9 |
| *Veillonella parvula*, AY995767 | 84.9 |
| *Megasphaera massiliensis*, JX424772 | 84.8 |
| *Anaeroglobus geminatus*, AF338413 | 84.8 |
| *Veillonella dispar*, AF439639 | 84.6 |
| *Veillonella montpellierensis*, AF473836 | 84.5 |
| *Desulfosporosinus acididurans*, HG316991 | 84.2 |
| *Dialister pneumosintes*, X82500 | 84.1 |
| *Dialister propionicifaciens*, AY850119 | 83.9 |
| *Allisonella histaminiformans*, AF548373 | 83.7 |
| *Negativicoccus succinicivorans*, FJ715930 | 83.7 |
| *Dialister invisus*, AY162469 | 83.2 |
| *Dialister succinatiphilus*, AB370249 | 82.9 |

**Supplementary Table S2 –** Substrate consumption and fermentation products from *Lucifera butyrica*.

| Substrate | Initial Concentration (mM) | Products (mM) | | | | | | | |
| --- | --- | --- | --- | --- | --- | --- | --- | --- | --- |
|  |  | **Acetate** | **Butyrate** | **Ethanol** | **Lactate** | **Malate** | **Propionate** | **Succinate** | **1,3-PDO** |
| Citrate | 10 | 33.87 | 2.70 | 4.66 |  | 0.12 | 1.5 |  |  |
| Glucose | 10 | 9.69 | 25.03 | 0.54 | 0.86 |  | 0.57 |  |  |
| Glycerol | 5 | 1.00 | 0.03 | 0.89 |  |  |  |  | 0.78 |
| Malate | 10 | 4.40 | 0.85 | 0.53 |  | 0.24 |  |  |  |
| Mannose | 10 |  | 1.55 |  |  | 0.35 |  | 0.08 |  |
| Pyruvate | 10 | 3.54 | 1.66 | 1.03 |  |  |  |  |  |
| Rhamnose | 10 | 4.19 | 4.54 |  |  |  | 2.45 |  |  |
| Serine | 5 | 3.15 |  |  |  |  |  |  |  |
| Xylose | 10 | 0.52 | 2.44 | 2.25 |  |  |  |  |  |
| Yeast Extract | 10 g L^-1^ | ? |  |  |  |  |  |  |  |
| Peptone | 10 g L^-1^ | 1.70 | 0.60 | 0.71 |  |  |  |  |  |

**Supplementary Table S3 –** Substrates consumption and products formation from the respiratory processes in *Lucifera butyrica* cultures.

| Electron Acceptor | Electron Donor | Electron Acceptor Consumption (mM) | Electron Donor Consumption (mM) | Products (mM) | | | | | |
| --- | --- | --- | --- | --- | --- | --- | --- | --- | --- |
|  |  |  |  | **Sulfide** | **Acetate** | **1,3-PDO** | **Lactate** | **Ethanol** | **Butyrate** |
|  | Glycerol | - | 3.23 | 0.75 | 1.03 | 0.86 |  | 0.55 | 0.15 |
| Elemental sulfur | Glycerol | ND | 2.64 | 2.26 | 0.73 | 0.34 |  |  |  |
| Thiosulfate | Glucose | 5.4 | 9.36 | 4.40 | 1.63 |  | 1.05 |  | 1.78 |
| DMSO | Glycerol | 1.87 | 2.93 |  | 1.01 | 0.88 |  |  |  |
| Iron | Glucose | ND | ND |  |  |  |  |  |  |

Supplementary Table S4 - Relative abundance (% of total) of cellular fatty acids of strain ALE^T^ and its phylogenetic closest relatives growing on glycerol. Data from this study.

| Fatty acids | Strain ALE^T^ | *Propionispora hippei* | *Propionispora vibrioides* |
| --- | --- | --- | --- |
| Saturated straight-chain | | | |
| *C_9:0_* | - | 0.38 | 0.45 |
| *C_10:0_* | - | 0.24 | 0.27 |
| *C_11:0_* | - | 13.45 | 14.67 |
| *C_12:0_* | 2.47 | - | - |
| *C_14:0_* | 5.25 | 1.02 | 0.82 |
| *C_15:0_* | - | 13.49 | 9.87 |
| *C_16:0_* | **22.66** | 2.81 | 2.87 |
| *C_17:0_* | - | 15.36 | 14.12 |
| *C_18:0_* | 3.59 | 0.61 | 0.66 |
| *C_19:0_* | - | 0.41 | 0.66 |
| Unsaturated straight-chain | | | |
| *C_15:1_ w8c* | - | 6.71 | 4.91 |
| *C_15:1_ w6c* | - | 0.26 | 0.26 |
| *C_16:1_ w9c* | **13.77** | 0.63 | 0.65 |
| *C_16:1_ w7c* | **13.01** | - | - |
| *C_16:1_ w5c* | - | 0.70 | 0.60 |
| *C_17:1_ w9c* | - | 6.30 | 4.75 |
| *C_17:1_ w8c* | - | 3.52 | 4.33 |
| *C_17:1_ w6c* | 6.00 | 1.84 | 1.75 |
| *C_18:1_ w9c* | - | 0.68 | 0.71 |
| *C_18:1_ w7c* | 3.93 | - | - |
| *C_18:1_ w5c* | - | 0.42 | 0.57 |
| *C_20:1_ w7c* | - | 1.20 | 0.86 |
| Hydroxy acids | | | |
| *C_11:0_ 3OH* | - | 2.88 | 4.06 |
| *C_12:0_ 3OH* | - | 0.37 | 0.44 |
| *C_15:0_ 3OH* | - | 0.27 | 0.31 |
| *Iso-C_13:0_ 3OH* | 7.17 | - | - |
| Saturated branched-chain | | | |
| *Iso-C_11:0_* | 4.26 | - | - |
| *Anteiso-C_13:0_* | - | 0.17 | 0.17 |
| *Iso-C_14:0_* | - | 0.82 | 0.90 |
| *Anteiso-C_15:0_* | - | 11.76 | 12.38 |
| *Iso-C_15:0_* | 7.44 | 0.46 | 0.43 |
| *Iso-C_16:0_* | - | 2.59 | 3.21 |
| *Anteiso-C_17:0_* | - | 1.51 | 1.51 |
| *Iso-C_17:0_* | 3.93 | - | - |
| *Iso-C_20:0_* | - | 0.54 | 0.52 |
| Unsaturated branched-chain | | | |
| *Iso-C_17:1_ w10c* | 6.53 | - | - |

Supplementary Table S5 – Genes involved in the metabolic pathways and molecular function of *Lucifera* *butyrica*. Note: The locus tag for the genes encoded in *Lucifera butyrica* is LUCI_*. To avoid repetition of the prefix along the text, the locus tags are represented by the specific identifier

| Description | EC | Locus tag |
| --- | --- | --- |
| 1,3-PDO | | |
| Pyruvate ferredoxin oxidoreductase alpha subunit | EC 1.2.7.1 | 3094 |
| Propanediol dehydratase large subunit | EC 4.2.1.28 | 0503 |
| Propanediol dehydratase medium subunit | EC 4.2.1.28 | 0504 |
| Propanediol dehydratase small subunit | EC 4.2.1.28 | 0505 |
| Propanediol dehydratase reactivation factor large subunit | - | 0506 |
| Propanediol dehydratase reactivation factor small subunit | - | 0507 |
| 1,3-propanediol dehydrogenase | EC 1.1.1.202 | 1837 |
| ATP:Cob(I)alamin adenosyltransferase | EC 2.5.1.17 | 0887 |
| Glycerol uptake facilitator protein | - | 0660 |
| Glycerol kinase | EC 2.7.1.30 | 0659 |
| Hypothetical protein | - | 4870 |
| Glycerol dehydrogenase | - | 4400 |
| Dihydroxyacetone subunit DhaK | EC 2.7.1.121 | 1901 |
| Dihydroxyacetone subunit DhaL | EC 2.7.1.121 | 1900 |
| Dihydroxyacetone subunit DhaM | EC 2.7.1.121 | 1899 |
| TCA cycle | | |
| Pyruvate ferredoxin oxidoreductase alpha subunit | EC 1.2.7.1 | 3094 |
| 2-oxoglutarate/2-oxoacid ferredoxin oxidoreductase subunit alpha | EC 1.2.7.3 | 4376, 1587, 2083 |
| Pyruvate carboxylase | EC 6.4.1.1 | 5107 |
| Dihydrolipoamide dehydrogenase | EC 1.8.1.4 | 0133, 1677, 3618, 4180 |
| Phosphoenolpyruvate carboxykinase (ATP) | EC 4.1.1.49 | 3947 |
| Citrate synthase | EC 2.3.3.1 | 0359 |
| Aconitate hydratase | EC 4.2.1.3 | 1546 |
| Isocitrate dehydrogenase (NAD+) | EC 1.1.1.41 | 2808 |
| Dihydrolipoamide dehydrogenase | EC 1.8.1.4 | 3618, 4180 |
| Succinyl-CoA:acetate CoA-transferase | EC 2.8.3.18 | 1562 |
| Fumarate reductase flavoprotein subunit | EC 1.3.5.4 | 0575 |
| Fumarate hydratase, class I | EC 4.2.1.2 | 0630 |
| Malate dehydrogenase | EC 1.1.1.37 | 2131 |
| Sulfate relay system | | |
| Cysteine desulfurase | [EC 2.8.1.7](https://www.kegg.jp/dbget-bin/www_bget?ec:2.8.1.7) | 1242 |
| Thiosulfate/3-mercaptopyruvate sulfurtransferase | EC 2.8.1.1 / 2.8.1.2 | 0570 |
| GTP 3',8-cyclase | EC 4.1.99.22 | 4422 |
| tRNA-uridine 2-sulfurtransferase | [EC 2.8.1.13](https://www.kegg.jp/dbget-bin/www_bget?ec:2.8.1.13) | 1240 |
| Molybdopterin adenylyltransferase | [EC 2.7.7.75](https://www.kegg.jp/dbget-bin/www_bget?ec:2.7.7.75) | 4425 |
| Molybdopterin-synthase adenylyltransferase | EC 2.7.7.80 | 4911 |
| Sulfur carrier protein ThiS adenylyltransferase | EC 2.7.7.73 | 1136 |
| Sulfite reductase, dissimilatory-type subunit gamma | EC 1.8.99.5 | 1126 |
| Sulfite reductase, dissimilatory-type subunit beta | EC 1.8.99.5 | 1128 |
| Resistance genes | | |
| Copper homeostasis | | |
| Copper-translocating P-type ATPase | EC 3.6.3.6 | 3071, 3935 |
| Multidrug resistance transporter | - | 4873 |
| Multicopper oxidase | EC 1.16.3.1 | 4187 |
| Cobalt-zinc-cadmium resistance | | |
| Cobalt-zinc-cadmium resistance protein | - | 1692, 2763, 2963, 2975 |
| Cobalt-zinc-cadmium resistance protein | - | 1700, 2101 |
| Transcriptional regulator, MerR family | - | 0108, 0153, 0163, 2633, 3905, 4895, 4973, 0101 |
| Zinc resistance | | |
| Response regulator of zinc sigma-54-dependent two-component system | - | 1031, 1930, 1938, 2953, 2955, 4071, 4072, 4079, 4080, 4146, 4421, 5023 |
| ZIP zinc transporter | - | 4845 |
| Arsenic resistance | | |
| Arsenical resistance operon repressor | - | 4106 |
| Arsenical resistance operon trans-acting repressor | - | 1662 |
| Arsenical pump-driving ATPase | - | 1661, 2676 |
| Arsenic efflux pump protein | - | 2859, 3114 |
| Arsenate reductase | EC 1.20.4.1 | 1669, 1685, 4104, 4171, 4174 |
| Arsenical-resistance protein ACR3 | - | 1667, 1687, 4172 |
| Cadmium resistance | | |
| Cadmium efflux system accessory protein | - | 2974, 3936 |
| Acid-related Resistance Mechanisms | | |
| Potassium-transporting ATPase | - | 2313, 2314, 2315 |
| Histidine kinase |  | 2311, 2312 |
| Glutaredoxin | - | 1706, 4105 |
| RecA | - | 1219 |
| MutS | - | 4052, 1208 |
| RecX | - | 1218 |
| UvrABC | - | 0694 |
| Oxalate formate antiporter | - | 3096 |
| Phosphate ABC transporter ATP-binding protein | - | 1612 |
| Amino acid ABC transporter ATP-binding protein | EC 3.6.3.27 | 0713, 2372, 2664 |
| Oxidative stress | | |
| Superoxide dismutase | EC 1.15.1.1 | 0236 |
| Phytochrome, two-component sensor histidine kinase | - | 0628 |
| Redox-sensitive transcriptional regulator | - | 1561 |
| Ferric uptake regulation protein FUR | - | 1248, 4923 |
| transcriptional regulator, Crp/Fnr family | - | 0240 |
| Alkyl hydroperoxide reductase subunit C-like protein | EC 1.11.1.15 | 1771 |
| Zinc uptake regulation protein ZUR | - | 3637 |
| Probable peroxiredoxin | EC 1.11.1.15 | 3079 |
| NADPH quinone oxidoreductase 2 | EC 1.6.5.2 | 2427, 2465 |
| Lactoylglutathione lyase | EC 4.4.1.5 | 2886 |
| Hydroxyacylglutathione hydrolase | EC 3.1.2.6 | 1250 |
| CoA-disulfide reductase | EC 1.8.1.14 | 1575 |
| Rubrerythrin | - | 2217, 3932, 4225 |
| Superoxide reductase | EC 1.15.1.1 | 3972 |
| Rubredoxin | EC 1.18.1.4 | 1096, 5138, 3460 |
| rubredoxin-oxygen oxidoreductase | EC 1.14.15.3 | 3763 |
| Beta-lactamase | | |
| Beta-lactamase | EC 3.5.2.6 | 4273 |
| Metal-dependent hydrolases of the beta-lactamase superfamily I | EC 3.1.4.55 | 1151 |
| Metal-dependent hydrolases of the beta-lactamase superfamily II | EC 3.1.4.55 | 3646, 4919 |
| Resistance to Vancomycin | | |
| Vancomycin B-type resistance protein VanW | - | 1193, 1591, 3786 |
| UDP-N-acetylglucosamine--N-acetylmuramyl-(pentapeptide) | EC 2.4.1.227 | 1030 |
| Phospho-N-acetylmuramoyl-pentapeptide-transferase | EC 2.7.8.13 | 2001 |
| UDP-N-acetylmuramoyl-tripeptide--D-alanyl-D-alanine ligase | EC 6.3.2.10 | 1439 |
| D-alanine-D-alanine ligase | EC 6.3.2.4 | 1438 |
| Alanine racemase | EC 5.1.1.1 | 1205 |
| Multidrug Resistance Efflux Pumps | | |
| RND efflux system, membrane fusion protein | - | 2092, 4220 |
| Type I secretion outer membrane protein | - | 2681 |
| Transcription regulator of multidrug efflux pump operon | - | 2454 |
| Multi antimicrobial extrusion protein (Na(+)/drug antiporter) | - | 1909, 2360, 3461, 4364 |
| Multidrug-efflux transporter, major facilitator superfamily | - | 3580, 3653, 4256, 4299, 4332 |
| Macrolide-specific efflux | - | 0216 |
| Macrolide export ATP-binding | - | 0217, 0218 |
| Acriflavin resistance protein | - | 1169, 0185, 3033, 3067, 3540, 4941 |
| Osmoregulation | | |
| Glycerol uptake facilitator protein | - | 1688, 2547, 0660 |
| Cold shock, CspA family of proteins | | |
| Cold shock protein CspA | - | 3440 |
| Cold shock protein CspC | - | 0712 |
| Heat shock dnaK gene cluster extended | | |
| Ribonuclease PH | - | 1744 |
| Phosphoesterase | - | 1746 |
| Nucleoside 5-triphosphatase RdgB | - | 1745 |
| Translation elongation factor LepA | - | 1318 |
| Hypothetical radical SAM family enzyme | - | 1247, 1317 |
| Ribosomal protein L11 methyltransferase | - | 1311 |
| Heat-inducible transcription repressor | - | 1316 |
| Chaperone protein DnaJ | - | 1312, 3156, 4305 |
| Heat shock protein GrpE | - | 1313 |
| Heat shock protein GrpE | - | 1314 |
| tmRNA-binding protein | - | 2810 |
| RNA polymerase sigma factor | - | 4545 |
| Carbon Starvation | | |
| Starvation sensing protein | - | 2540 |
| Starvation sensing protein | - | 2538, 4915 |
| Carbon starvation protein A | - | 1162, 2302, 3225 |
| ferredoxin NADP oxidoreductase | EC 1.18.1.2 | 1587, 1907, 2081, 2596, 2903, 2905, 3092, 3164, 3176, 4378 |
| cysteine/aspartate aminotransferase | EC 2.6.1.1 | 0413, 0613, 2048, 2196, 3978, 4902 |
